# Fast Broad-Spectrum Staining and Photodynamic Inhibition of Pathogenic Microorganisms by a Water-Soluble Aggregation-Induced Emission Photosensitizer

**DOI:** 10.3389/fchem.2021.755419

**Published:** 2021-11-02

**Authors:** Qi Zhou, Xiaoming Lyu, Bing Cao, Xueping Liu, Jing Liu, Jiarui Zhao, Siyu Lu, Meixiao Zhan, Xianglong Hu

**Affiliations:** ^1^ MOE Key Laboratory of Laser Life Science and Institute of Laser Life Science, College of Biophotonics, South China Normal University, Guangzhou, China; ^2^ Guangdong Provincial Key Laboratory of Laser Life Science and Guangzhou Key Laboratory of Spectral Analysis and Functional Probes, College of Biophotonics, South China Normal University, Guangzhou, China; ^3^ Department of Laboratory Medicine, The Third Affiliated Hospital, Southern Medical University, Guangzhou, China; ^4^ Green Catalysis Center and College of Chem, Guangzhou, China; ^5^ Zhuhai Precision Medical Center, Zhuhai People’s Hospital, Zhuhai Hospital Affiliated with Jinan University, Jinan University, Zhuhai, China

**Keywords:** aggregation-induced emission, photosensitizer, fast staining, photodynamic therapy, pathogenic microorganisms

## Abstract

Pathogenic microorganisms pose great challenges to public health, which is constantly urgent to develop extra strategies for the fast staining and efficient treatments. In addition, once bacteria form stubborn biofilm, extracellular polymeric substance (EPS) within biofilm can act as protective barriers to prevent external damage and inward diffusion of traditional antibiotics, which makes it frequently develop drug-resistant ones and even hard to treat. Therefore, it is imperative to develop more efficient methods for the imaging/detection and efficient inhibition of pathogenic microorganisms. Here, a water-soluble aggregation-induced emission (AIE)-active photosensitizer TPA-PyOH was employed for fast imaging and photodynamic treatment of several typical pathogens, such as *S. aureus*, methicillin-resistant Staphylococcus aureus, *L. monocytogenes*, *C. albicans*, and *E. coli*. TPA-PyOH was non-fluorescent in water, upon incubation with pathogen, positively charged TPA-PyOH rapidly adhered to pathogenic membrane, thus the molecular motion of TPA-PyOH was restricted to exhibit AIE-active fluorescence for turn-on imaging with minimal background. Upon further white light irradiation, efficient reactive oxygen species (ROS) was *in-situ* generated to damage the membrane and inhibit the pathogen eventually. Furthermore, *S. aureus* biofilm could be suppressed *in vitro*. Thus, water-soluble TPA-PyOH was a potent AIE-active photosensitizer for fast fluorescent imaging with minimal background and photodynamic inhibition of pathogenic microorganisms.

## Introduction

Pathogenic microorganisms, especially drug-resistant bacteria, are posing more and more severe threat to human health in last decades. (Bartell et al., 2019; Kang et al., 2019) According to the World Health Organization, bacterial infection caused highest death rate in less developed countries in the past 15 years. (Wang Y. et al., 2020) Due to the high infectivity and mortality of bacteria, bacterial infection threats the public health and economic development all over the world. The use of antibiotic, such as penicillin and colistin, has saved millions of people and has achieved periodical success. (Yu et al., 2021) However, antibiotics are double-aged swords. Long-time use and overuse of traditional antibiotics have led to multidrug-resistance bacteria as far as superbug. Apart from that, extracellular polymeric substances form biofilm and protect bacteria from treating and external environments. (Liu et al., 2016; Cao et al., 2020) Due to the presence of biofilm, bacteria become more and more strong to result in more challenging public health issues.

To tackle the dilemma of antibiotics, many efforts have been paid to develop alternative antibacterial agents and materials with extra inhibition mechanisms. (Lakemeyer et al., 2018) With the rapid progress of medicine and interdisciplinary, new antibacterial systems have received more and more attention. (Xi et al., 2019; Cui et al., 2020) Several antimicrobial systems with diverse modalities have been designed to eliminate or inhibit pathogenic microorganisms, such as inorganic antibacterial materials, (Qiao et al., 2020), antimicrobial peptides (AMPs), (Chen H. et al., 2018; Yang et al., 2020) hydrogels, (Wang et al., 2019; Zhu et al., 2019; Tian et al., 2020), polymeric antimicrobials, (Cao et al., 2018; Lu et al., 2018), antibacterial coatings, (Wang B. et al., 2018; Wei et al., 2019), phototherapeutic antibacterial systems, (Qing et al., 2019; Li Y. et al., 2020; Wang Y. et al., 2020; Guo et al., 2020; He et al., 2020; Yuan et al., 2020; Zhao et al., 2020) etc.

As a novel antibacterial method, photodynamic therapy (PDT) has attracted more and more attention for bacterial inhibition. (Liu et al., 2019; Xiao et al., 2020) Compared with traditional antibiotics, the advantages of PDT are prominent, such as non-invasive nature, favorable spatiotemporal control, negligible drug resistance, and low systemic toxicity. (Yao et al., 2020; Qi et al., 2021) Photosensitizers can transfer photon energy to surrounding oxygen molecules to produce ROS eventually for therapeutic applications in typical PDT processes. (Dai et al., 2020) In terms of photodynamic inhibition of pathogenic microorganisms, the exploration of photosensitizers in PDT to produce ROS for antibacterial therapy and localized pathogen elimination has made great progress, (Hu et al., 2019), but traditional photosensitizers inevitably suffer from well-known aggregation-caused quenching (ACQ) effect, which has caused serious compromise of fluorescence emission and ROS generation in aggregation state. (Tang, 2020; Xu et al., 2020) Most traditional photosensitizers are hydrophobic, their self-assembled aggregates and subsequent interaction with cells usually lead to fluorescence quenching and decreased ROS generation. In the past 2 decades, the proposed AIE concept has shaded light on diverse fields as well as novel AIE-active photosensitizers for biological applications, (He et al., 2019; Kang et al., 2020), which have been widely applied for *in vitro* and *in vivo* detection and theranostics. (Pandey and Chakravarthy, 2021) AIE-active photosensitizers show strong fluorescence emission and ROS generation in aggregation state, which benefits from the mechanism of restriction of intramolecular motion. (Hu F. et al., 2018; Li Q. et al., 2020) In addition, the exploration of water-soluble AIE-active photosensitizers is favorable to reduce imaging background and promote the resultant theranostic performances in restricted aggregates. (Wang D. et al., 2018) The generated ROS can oxidize DNA, RNA and lipids of bacterial membrane and cells, and lead to bacterial destruction. (Chen S. et al., 2018; Li et al., 2021).

**FIGURE 1:**
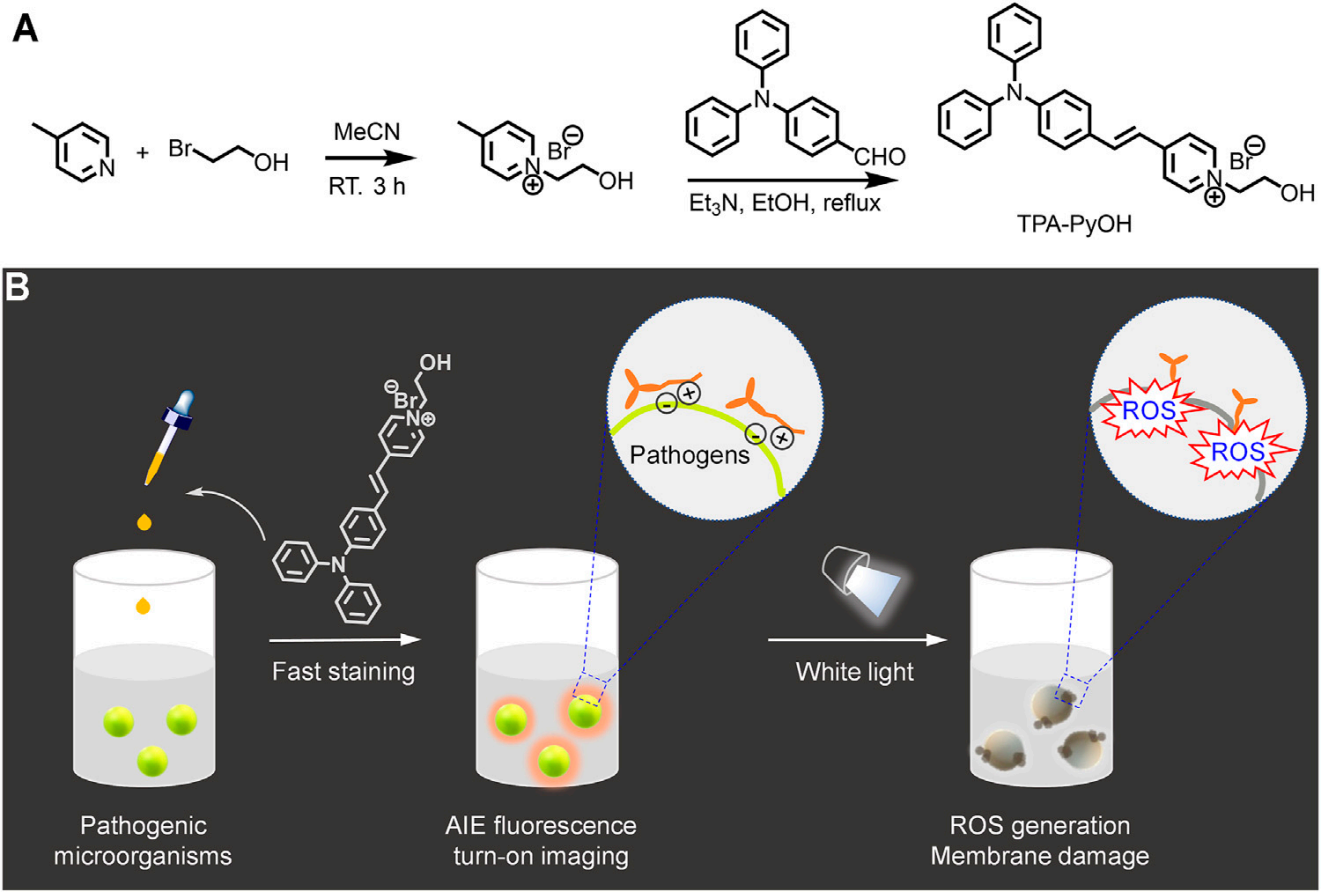
Schematic illustration for **(A)** the synthesis of a water-soluble aggregation-induced emission (AIE)-active photosensitizer, TPA-PyOH. **(B)** Its application for fast staining and imaging of pathogenic microorganisms. Upon white light irradiation, reactive oxygen species (ROS) was *in situ* generated to inhibit pathogens efficiently.

In this work, an AIE-active photosensitizer, TPA-PyOH, was developed for fast pathogen imaging and photodynamic inhibition (Figure 1). TPA-PyOH had a quaternary ammonium group and a terminal hydroxyl unit, possessing excellent water solubility, so its biological application was widely applicable with favorable working concentration range. More importantly, it demonstrated good antibacterial property via *in vitro* analysis, including not only Gram-positive bacteria, such as *S. aureus*, methicillin-resistant *Staphylococcus aureus* (MRSA), and *Conocytogenes*, but also Gram-negative *E. coli*. Apart from that, *C. albicans*, a fugus, was also inhibited effectively by the photodynamic effect of TPA-PyOH. After that, the *S. aureus* biofilm could be also inhibited. In addition, as a proof-of-concept, TPA-PyOH was also employed for wash-free cell imaging, which could potentially further promote its extra theranostic applications.

## Experimental

### Chemicals and Materials

4-methylpyridine and 2-bromoethan-1-ol were purchased from Aladdin. Dichloromethane (DCM) was distilled over CaH_2_. Methyl sulfoxide (DMSO), *N, N*-dimethylformamide (DMF), ethanol, and all other reagents were purchased from Sinopharm Chemical Reagent Co., Ltd., and used as received. SYTO 9 and propidium iodide (Invitrogen LIVE/DEAD®BacLight Bacterial Viability Kit, L7012) was purchased from Thermo Fisher Scientific. Water used in this study was deionized with a Milli-QSP reagent water system (Millipore). *S. aureus* (ATCC 6538) and MRSA (ATCC 43300) were used as Gram-positive bacteria. *E. coli* (ATCC 25922) was used as Gram-negative bacteria, and *C. albicans* (ATCC 10231) was used as a representative fungus.

### Synthesis of TPA-PyOH

As shown in Figure 1A, TPA-PyOH was synthesized via facile two steps. (Wang Y.-L. et al., 2020). Its structure was characterized by ^1^H NMR spectrum (Supplementary Figure S1).

### Characterization

UV-vis spectra were obtained by an UV-2600 ultraviolet and visual spectrophotometer (Shimadzu, Japan). Fluorescence spectra were investigated by an FL-1000 Steady State and Transient State Fluorescence Spectrometer (Edinburgh Instruments Ltd., United Kingdom). CLSM imaging was performed on a confocal laser scanning microscope (Carl Zeiss LSM 880 META, Germany).

### Single Oxygen (^1^O_2_) Detection

Typically, ABDA (20 μl stock solution, 5 mM) was added to the dispersion of TPA-PyOH, and finally diluted into 2 ml by deionized water. The mixed dispersion was irradiated by white light for different time and the absorption intensity of ABDA at 378 nm was recorded.

### Confocal Laser Scanning Microscopy Imaging

The interaction of TPA-PyOH with pathogenic microorganisms was observed by confocal laser scanning microscopy (CLSM) imaging. Typically, a single colony of microorganism was inoculated in Luria-Bertani (LB) broth at 37°C and shaking overnight. After 12 h, the suspension was centrifuged and washed with PBS buffer for three times, then the suspension was adjusted to OD600 = 0.1 with PBS buffer. The microorganism dispersions were co-incubated with TPA-PyOH (2.5 μM or 5 μM) or PBS for 5 min at 37°C, respectively. Then the dispersion was centrifuged and washed with deionized water for three times. Finally, the residual microorganism cells were imaged by the CLSM system.

### *In Vitro* Antibacterial Activity

The MIC values of TPA-PyOH against *S. aureus*, MRSA, *E. coli*, and *C. albicans* were determined by standard procedures. (Li et al., 2014) Typically, a single colony of microorganism was inoculated in Luria-Bertani (LB) broth and kept shaking at 37°C overnight, then the suspension was diluted to OD600 = 0.001 (*S. aureus,* MRSA, *and E. coli*) or OD600 = 0.1 (*C. albicans*) with LB broth. The aqueous solution of TPA-PyOH was adjusted to a series of two-fold dilutions with deionized water. Microorganism suspension (100 μl) was added to the diluted TPA-PyOH dispersion (100 μl) at 96-well plates, and the mixture was incubated at 37°C for 30 min. The mixture was subjected to white light for 20 min or at dark, respectively. After growing at 37°C for 24 h, the turbidity of microorganism with TPA-PyOH or PBS buffer was recorded by OD at 600 nm using a microplate reader (infinite 200 pro, Tecan).

### Zeta Potential Analysis

The dispersions of *S. aureus* and *E. coli* were incubated overnight, respectively, then diluted to OD = 0.1. The bacteria dispersion was then stained with TPA-PyOH for 10 min, the zeta potential value was finally determined by a Malvern Zetasizer.

### Formation of *S. aureus* Biofilm

Typically, single colony of *S. aureus* was inoculated in LB broth at 37°C. After shaking overnight, the bacterial suspension was adjusted to OD600 = 0.02, then added into 96-well plates. The 96-well plate with bacterial suspension was cultivated at 37°C for 72 h. The medium was refreshed every 24 h and planktonic bacteria were removed by PBS washing. Eventually, the *S. aureus* biofilm was formed in the bottom of 96-well plates.

### *In Vitro* Biofilm Inhibition Assay

*S. aureus* biofilm was formed on 96-well plates according to the protocol described above. The LB broth was removed, and the biofilm was washed by PBS buffer for two times to wash away the planktonic bacteria. The obtained biofilm was incubated with PBS or TPA-PyOH (100 μM) at 37°C for 2 h at dark. After incubating for 2 h, the mixture was treated with white light irradiation (∼38.6 mW/cm^2^, 20 min) or kept at dark in the whole process, then the residual biofilms were rinsed with PBS buffer for three times. After that, PBS (200 μl) was added into each well and treated with ultrasonication for 10 min to disperse the residual biofilm. Finally, the bacteria colony from the residual biofilm was calculated by Mueller-Hinton (MH) agarose plate analysis.

### Live/Dead Staining Assay

*S. aureus* were cultured to form biofilm on a poly (methyl methacrylate) (PMMA) slide (10 mm × 10 mm × 0.2 mm) in 24-well microtiter plates at 37°C. After 72 h, free bacteria were rinsed with sterile PBS buffer. Then the obtained biofilms were incubated with PBS or TPA-PyOH for 2 h at 37°C. After irradiation by white light or at dark, the residual biofilm was washed by PBS buffer and stained by the LIVE/DEAD BacLight bacterial viability kit. Finally, 3D confocal images were obtained on the CLSM system. Bacteria stained with green and red were considered to be living and dead, respectively.

### Cell Viability Assay

MTT assay was used to measure potential cytotoxicity of TPA-PyOH. Human pulmonary epithelial cells (A549 cells) were seeded in 96-well plates. After culture for 24 h at 37°C in a humidified incubator (5% CO_2_), the culture medium was refreshed and added with TPA-PyOH with different concentration and co-incubated for 24 h. Then the culture milieu was refreshed, and MTT agent was added into the 96-well plates to incubate for another 4 h. Then, the milieu was removed, and DMSO was added into the plates. The absorbance intensity at 570 nm was measured by a microplate reader.

### Hemolytic Analysis

Hemolysis was evaluated by rat red blood cells (RBCs). RBCs were collected by centrifugation (1,000 rpm, 20 min). After washing with PBS buffer for three times, the dispersion of RBCs (100 μl) was added into diverse samples with different contents (900 μl) and incubated at 37°C for 4 h. Finally, the mixture was centrifuged at 1,000 rpm for 5 min. The supernatant was collected and the absorbance at 540 nm was recorded by a microplate reader.

## Results and Discussion

### Characterization of TPA-PyOH

**FIGURE 2:**
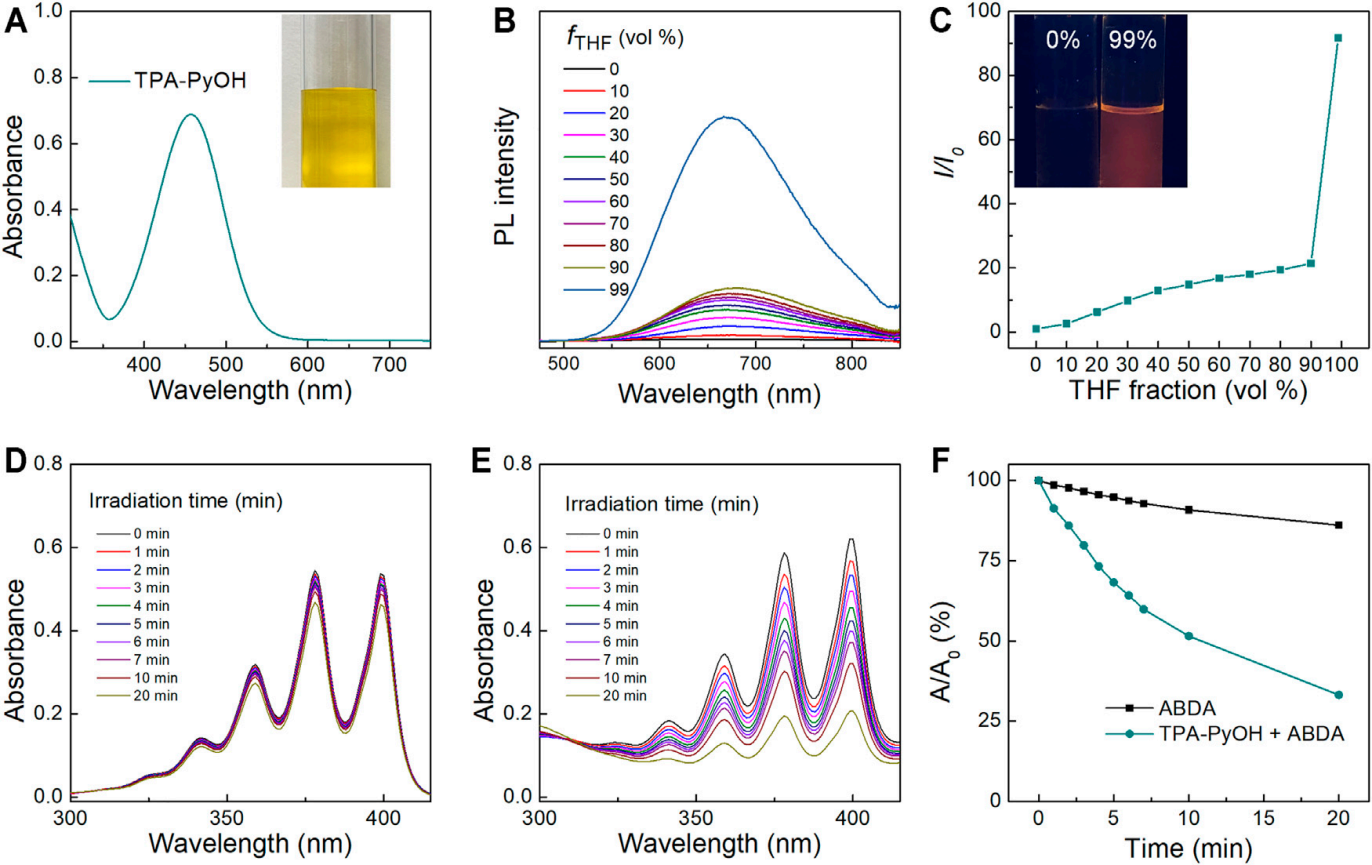
**(A)** UV-vis absorption spectrum of TPA-PyOH and its representative photograph of aqueous solution. **(B)** PL spectra of TPA-PyOH (10 mM) in water/THF mixtures with different THF volume fractions (*f*_THF_). **(C)** The plot of the emission maximum and the relative emission intensity (*I/I*_*0*_) versus the composition of the water/THF mixtures of TPA-PyOH. Inset: fluorescence photographs of TPA-PyOH in the pure water and in water/THF mixtures with 99% THF fractions under 365 nm UV irradiation. **(D)** Absorption spectra of ABDA (50 μM) upon white light irradiation for diverse durations. **(E)** Absorption spectra of ABDA in the presence of TPA-PyOH (20 μM) upon white light irradiation for diverse durations. **(F)** Plot of the relative absorbance of ABDA with white light irradiation under different conditions (A_0_ and A are the absorbance of ABDA at 378 nm before and after irradiation).

The absorbance peak of TPA-PyOH in water located at ∼457 nm (Figure 2A), the excitation of TPA-PyOH was not in ultraviolet region, thus avoiding its negative effects for biological applications. Typical AIE curve of TPA-PyOH was performed in diverse solvent fractions using water as a good solvent and tetrahydrofuran (THF) as a poor solvent. PL spectra of TPA-PyOH in water/THF mixtures with different THF volume fractions (*f*_THF_) were determined accordingly (Figure 2B). The emission maximum located at around 660–680 nm, along with the THF fraction gradually increases to 90%, the emission intensity increased gradually. However, when the THF fraction reached from 90 to 99%, the emission intensity increased dramatically and the emission peaks of it was ∼92 times higher than that in pure water solution. The insert fluorescent image of sample at 99% exhibited remarkable orange red fluorescence compared to its pure aqueous solution. The AIE cure of TPA-PyOH definitely indicated its AIE characteristics (Figure 2C), which was well consistent with some reported AIE systems. (Li et al., 2018; Wang et al., 2020b).

### *In Vitro* ROS Generation

The ROS generation capacity of TPA-PyOH was evaluated accordingly. Cytotoxic ROS in PDT can be generated by both type I and type II mechanisms, which produces superoxides and single oxygen (^1^O_2_), respectively (Yang et al., 2020). 9,10-Anthracenediyl-bis(methylene) dimalonic acid (ABDA) was used as indicator of ^1^O_2_. The absorbance of ABDA had no obvious decrease upon white light irradiation with different durations (Figure 2D). However, the absorption spectra of the mixture of ABDA and TPA-PyOH was changed significantly upon the same treatments (Figure 2E). Relative absorbance of ABDA at 378 nm showed that the photodynamic process of TPA-PyOH had remarkable ability to produce ^1^O_2_ (Figure 2F) (Zheng et al., 2020).

### Fast Imaging of Pathogenic Microorganisms and Wash-Free Cell Staining

**FIGURE 3:**
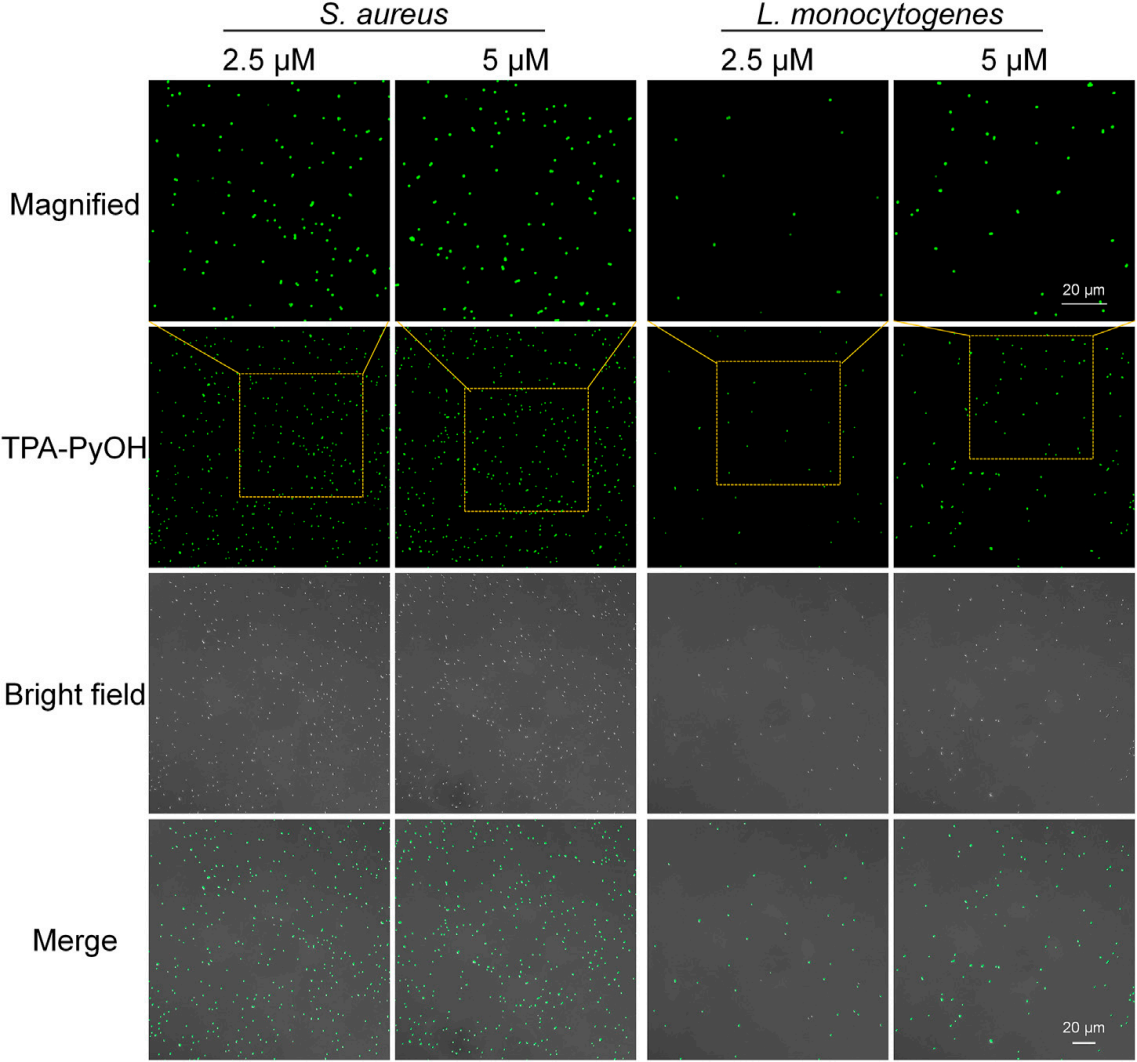
CLSM images (40×) of *S. aureus* and *L. monocytogenes* after staining with TPA-PyOH at diverse content for 5 min. TPA-PyOH was excited at 488 nm, and the emission range was collected at 520–670 nm. Green pseudo color is used to represent fluorescent signal from TPA-PyOH channel.

Aggregation-induced emission luminogen (AIEgen) molecular probes have intensive fluorescence emission upon aggregation arised from the mechanism of restriction of intramolecular motion. (Hu F. et al., 2018) The unique light-up fluorescence of AIEgens with excellent photostability has made them popular in fluorescence imaging in biomedicine. (He et al., 2020) Based on this, *in vitro* fluorescence imaging of microbes was evaluated by typical CLSM imaging. *S. aureus* and *L. monocytogenes* were selected as two Gram-positive bacterial models to explore their interaction behavior with TPA-PyOH. As shown in Figure 3, *S. aureus* and *L. monocytogenes* both showed obvious fluorescent signal after incubating TPA-PyOH for only 5 min. The working content of TPA-PyOH was also examined at 2.5 and 5 μM, respectively. There was no significant imaging difference between these two different concentrations. The native fluorescence of *S. aureus* was minimal in the absence of TPA-PyOH (Supplementary Figure S2).

Furthermore, Gram-positive *L. monocytogenes* is a significant threat in food industry and daily life. It is an opportunistic pathogen with a high fatality rate in susceptible populations. (Dygico et al., 2021) It can grow under adverse conditions in food production environment. Therefore, it is important to improve the detection efficiency of *L. monocytogenes*. In this work, the fast staining of *L. monocytogenes* was achieved upon only 5 min incubation with TPA-PyOH. Obviously, with the concentration improved from 2.5 to 5 μM, the bacterial fluorescence intensity increased slightly, thus it provided a new method for rapid staining and imaging of *L. monocytogenes.* The native fluorescence of *L. monocytogenes* was minimal in the absence of TPA-PyOH (Supplementary Figure S3).

**FIGURE 4:**
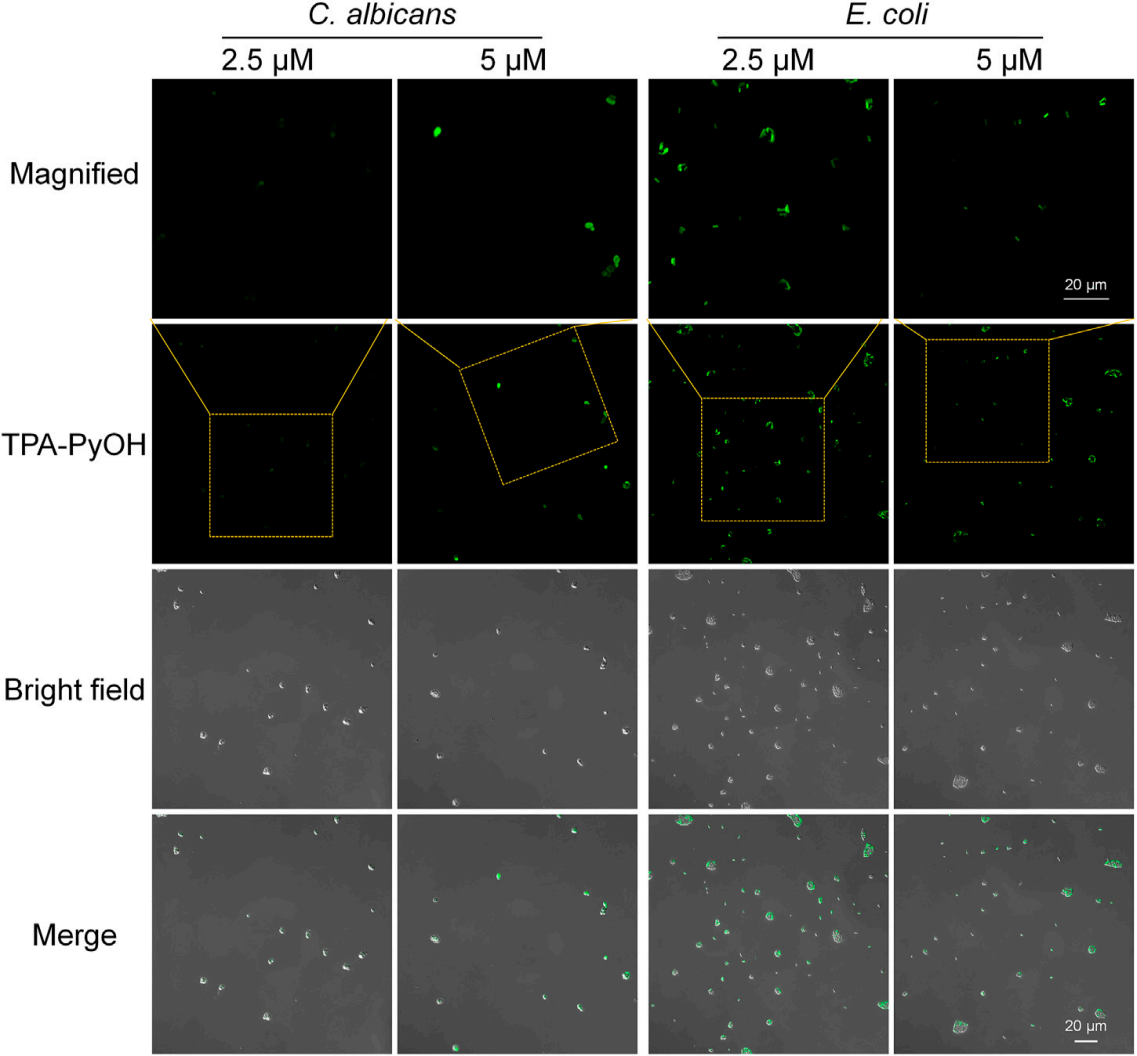
CLSM images (40×) of *E. coli* and *C. albicans* after staining with TPA-PyOH for 5 min. TPA-PyOH was excited at 488 nm, and the emission range was collected at 520–670 nm. Green pseudo color is used to represent fluorescent signal from TPA-PyOH channel.

After that, *E. coli* was employed as a representative Gram-negative bacterium for the imaging examination of TPA-PyOH. As presented in Figure 4, upon incubation for 5 min, the fluorescent intensity of *E. coli* was slightly weak. The result was speculated from the difference of their bacterial membrane compared with Gram-positive bacteria. Gram-negative bacteria are well-known to be intrinsically resistant to many antibiotics due to the permeability barrier that is provided by their unique cell envelope. (Hu X. et al., 2018) This envelope consists of an outer membrane (OM) and inner membrane (IM), which are separated by a periplasmic space. (Masi et al., 2017) The special structure of Gram-negative bacteria made TPA-PyOH slightly difficult to attach and diffuse in the membrane, but the short-time staining of typical Gram-negative bacteria was still discernable and effective. Similarly, the native fluorescence of *E. coli* was minimal in the absence of TPA-PyOH (Supplementary Figure S4). In addition, the dispersion of *S. aureus* and *E. coli* was incubated with TPA-PyOH for 10 min, respectively, (Supplementary Figure S5). The charge properties of these two bacteria kept negative, which primarily resulted from sufficient adhesion and inward diffusion of positively charged TPA-PyOH with the negative bacterial membrane.

Notably, *C. albicans* was also examined as an example of fungi. Upon staining with the same short duration, the concentration increasing of TPA-PyOH could further brighten the fluorescent images of *C. albicans* (Figure 4 and Supplementary Figure S6). The fast staining and imaging of *C. albicans* has been a challenge in biomedicine due to the presence of protective cell wall with complex eukaryotic cell structure. Slightly enhanced treating content of TPA-PyOH was favorable to achieve fast staining and fluorescent imaging of *C. albicans*.

Apart from the fast imaging of pathogenic microorganisms, TPA-PyOH was also interrogated for the staining of mammalian cells. Inspired from its minimal fluorescent background in pure water, wash-free staining of TPA-PyOH was performed for EMT6 cells and observed by CLSM imaging. (Huang et al., 2021). EMT6 cells were incubated with TPA-PyOH for 4 h and imaged directly without extra washing (Supplementary Figure 7), the fluorescence intensity of the cells was remarkable for facile observation, in which the fluorescence background was undetectable. This result agreed well with the AIE characteristic of water-soluble TPA-PyOH. In addition, the cytotoxicity of TPA-PyOH towards EMT6 cells was minimal at dark at the content of 5 μM (Supplementary Figure S8).

### *In Vitro* Photodynamic Inhibition of Pathogenic Microorganisms

**FIGURE 5:**
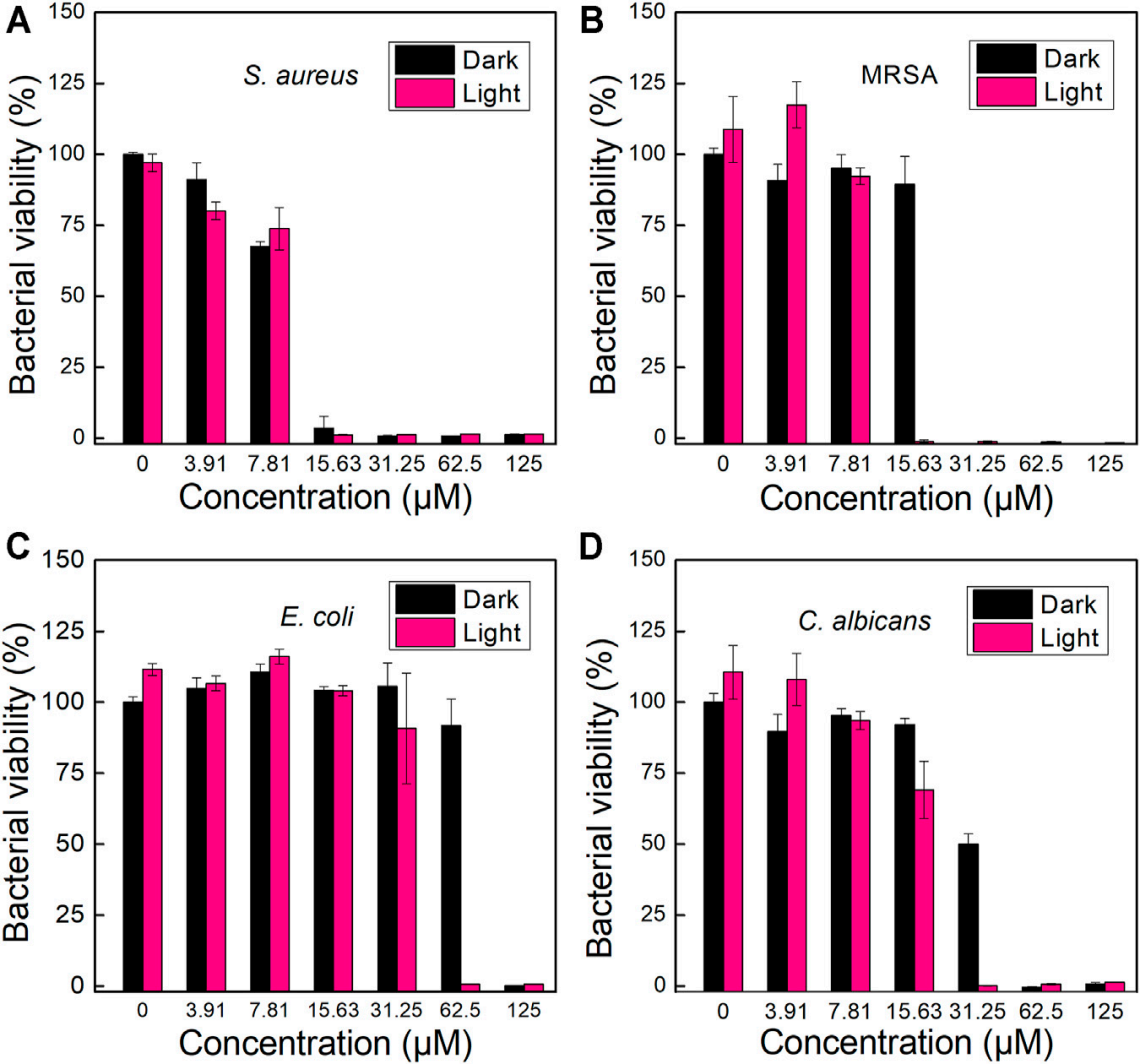
Growth inhabitation of **(A)** *S. aureus*, **(B)** MRSA*,* **(C)** *E. coli* and **(D)** *C. albicans* on 96-well plates after incubation with different dosage of TPA-PyOH.

Since TPA-PyOH could interact and diffuse in pathogenic microorganisms or probably accumulate on the cell surface, the ROS produced by the AIEgen could attack the microbe upon white light irradiation. Inspired by efficient fluorescence imaging and ROS production of TPA-PyOH, *in vitro* antimicrobial ability of TPA-PyOH was further evaluated in subsequent section. Using a microbroth dilution protocol, the antimicrobial activities of TPA-PyOH were evaluated against clinically representative pathogentic microorganisms, including *S. aureus*, MRSA, *E. coli*, and *C. albicans.* The turbidity of microbes upon diverse treatments was recorded by the OD values at 600 nm (Figures 5A–C). The killing effect of TPA-PyOH on Gram-positive *S. aureus* and MRSA was stronger than that of Gram-negative *E. coli*. It mainly resulted from the membrane structure of Gram-negative bacteria that probably inhibit or delay the adhesion and entry of the AIEgen, and some of the internalized molecules were even expelled by an efflux pump. (Masi et al., 2017) Herein, for Gram-negative bacteria, extracellular AIEgens were less effective in killing bacteria due to their less potency to enter the cells as well as limited action range and lifetime of ROS in subsequent PDT, which resulted in the compromised bacterial inhibition efficiency by TPA-PyOH. On the other hand, even in the dark condition, TPA-PyOH also showed moderate inhibition towards the Gram-positive bacteria. The PDT treatment of TPA-PyOH was determined to be effective towards *C. albicans* was also effective (Figure 5D).

**FIGURE 6:**
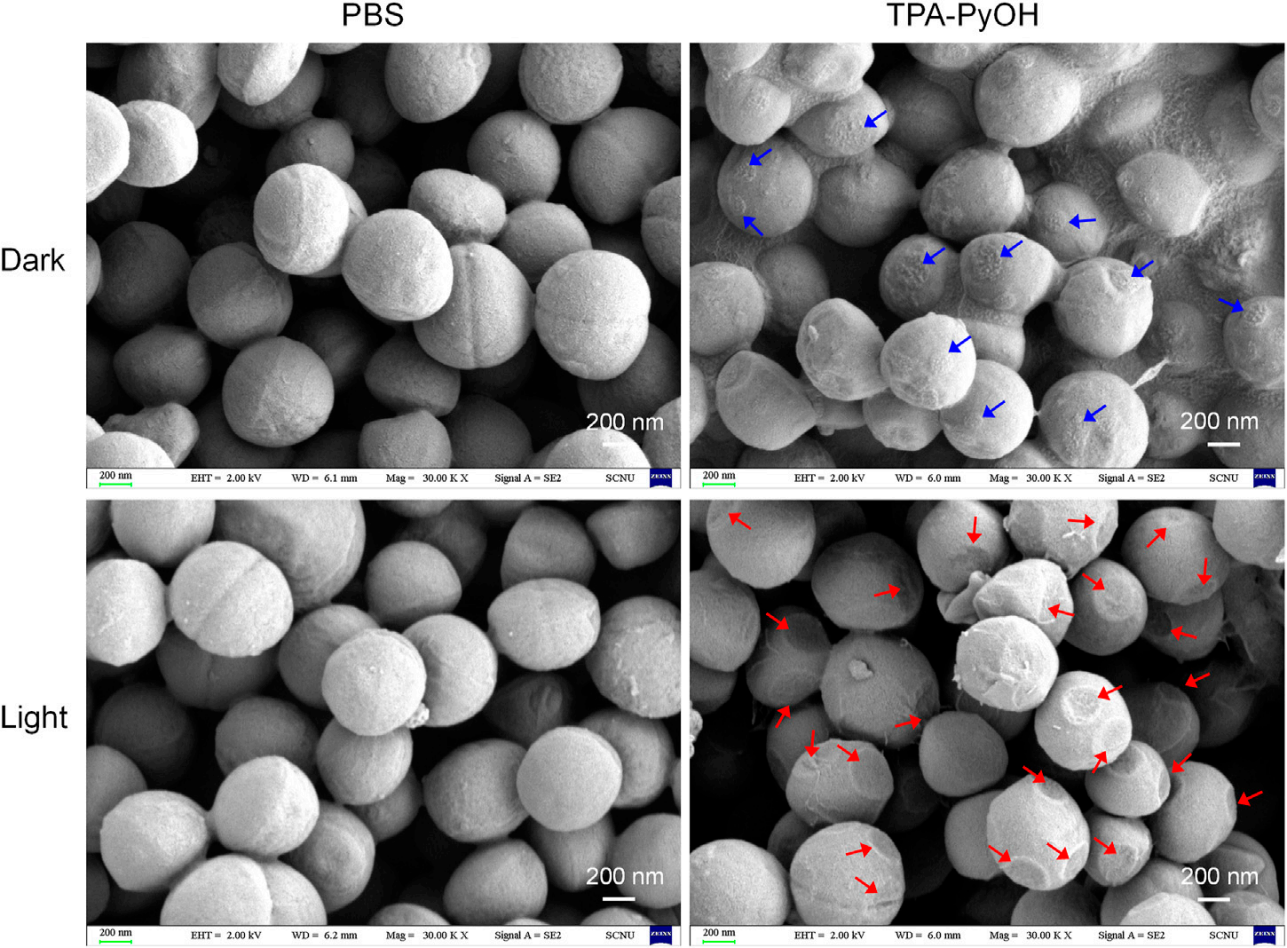
SEM images of *S. aureus* upon diverse treatments. The blue arrows indicated probable adhesion sites of TPA-PyOH with the bacteria. The red arrows indicated the obvious damage sites on the membrane upon photodynamic treatments.

The morphology of bacteria upon PDT of TPA-PyOH was further observed by SEM analysis, which could directly visualize subtle change of bacterial morphology for *S. aureus* upon diverse treatments (Figure 6). The untreated *S. aureus* cells displayed almost intact surface, which was comparable to the group with only white light irradiation. For the bacteria incubated with TPA-PyOH, bacterial cells were obviously coated with many small patches, which were indicated by blue arrows in the image, probably resulting from the adhesion and fusion of TPA-PyOH as well as co-assembly with the membrane. It provided favorable condition for *in-situ* ROS generation in the membrane in subsequent photodynamic treatment due to limited ROS damage range. (Zhang et al., 2019) Upon further white light irradiation, as noted by red arrows in the image, the bacteria membrane collapsed at the patching sites arised from TPA-PyOH accumulation and *in-situ* ROS damage. These direct observations from SEM analysis demonstrated the efficient PDT potency of TPA-PyOH in bacterial inhibition.

### *In Vitro* Biofilm Inhibition Analysis

**FIGURE 7:**
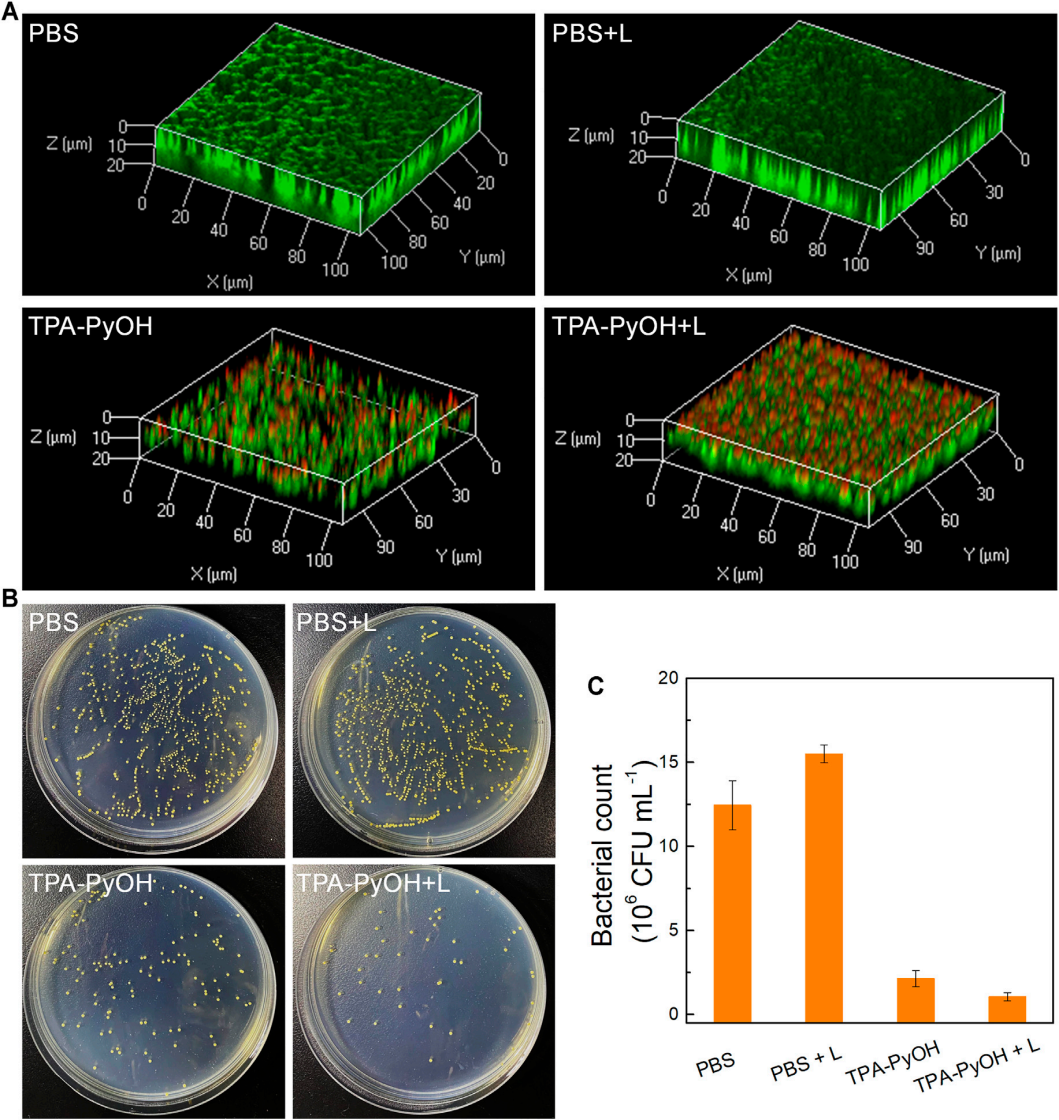
**(A)** The merged fluorescence images of *S. aureus* biofilms upon diverse treatments. The whole biofilm was co-stained by SYTO-9 (green channel) and PI (red channel); **(B)** Representative plate photographs of *S. aureus* colonies upon diverse treatments; **(C)** Number of viable *S. aureus* bacterial count after different treatments.

As a proof-of-concept, the inhibition effect of TPA-PyOH towards established biofilm was evaluated using *S. aureus* biofilm as a model. (Wang C. et al., 2020) Firstly, LIVE/DEAD staining assay was performed by 3D CLSM imaging for all biofilm samples after different treatments. As shown in Figure 7A, live bacteria and dead bacteria were noted in green (SYTO-9) and red fluorescence color (PI), respectively. Compared with other groups, the group of TPA-PyOH with white light irradiation displayed stronger red fluorescence in the biofilm. The group of TPA-PyOH without white light irradiation also displayed weak red fluorescence, suggesting moderate native inhibition effect of TPA-PyOH. These results indicated that the PDT of TPA-PyOH could inhibit biofilm to some extent. Efficient treating and eradication of the bacteria in deep biofilm was still challenging due to the protection of EPS. (Cao et al., 2020; Wang Y. et al., 2020) After that, the biofilm inhibition efficiency of TPA-PyOH was quantitatively examined, broth dilution method was used to calculate the residual bacteria after ultrasound. Representative photographs of plates with residual *S. aureus* biofilm after each treatment were shown in Figures 7B,C. The group of TPA-PyOH with white light irradiation exhibited most apparent killing effect for the *S. aureus* biofilm.

### Biocompatibility Analysis

**FIGURE 8:**
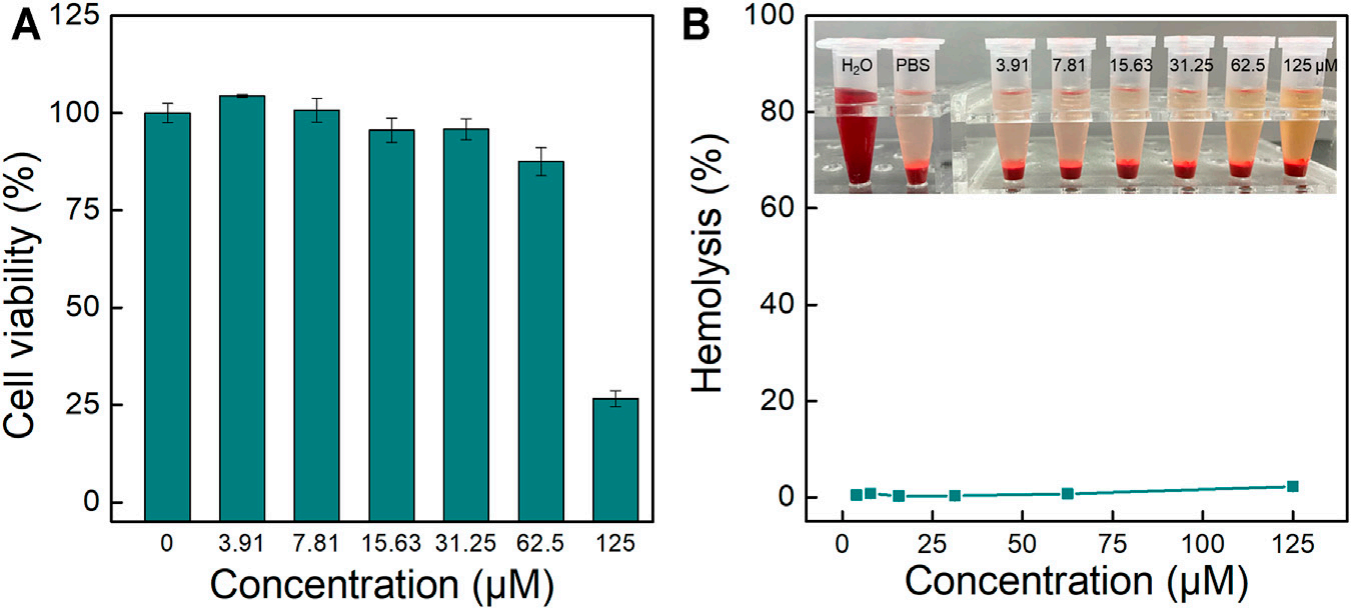
**(A)** *In vitro* cytotoxicity analysis for TPA-PyOH upon incubation with A549 cells for 24 h; **(B)** Typical hemolytic analysis of TPA-PyOH against mice red blood cells. Inset: Representative images for the samples upon diverse treatments in hemolytic analysis.

Then the biocompatibility of TPA-PyOH was evaluated, including cytotoxicity and hemolysis. The cell survival rate was observed to be ∼87.5% when the concentration of TPA-PyOH was up to ∼62.5 μM (Figure 8A). The result suggested that the cytotoxicity of AIEgen was minimal to mammalian cells. In addition, the hemolysis of TPA-PyOH was analyzed typically (Figure 8B). The hemolysis ratio was minimal for TPA-PyOH, determined to be <1%, as the content of increased from 0 to 62.5 μM. The AIEgen only caused <3% red blood cell hemolysis at a high content up to 125 μM. These results were also supported by photographs of different concentrations of samples incubated with red blood cells (Figure 8B insert). These results verified the acceptable biocompatibility of TPA-PyOH for promising biological applications.

## Conclusion

In summary, a water-soluble AIE-active photosensitizer, TPA-PyOH, was developed for fast imaging and photodynamic inhibition of pathogenic microorganisms. TPA-PyOH could fast stain and image several typical pathogens, such as *S. aureus*, MRSA, *L. monocytogenes*, *C. albicans*, and *E. coli*. The effective working concentration of TPA-PyOH could be as low as ∼2.5 μM to achieve favorable performance with minimal background interference. Upon white light irradiation, *in-situ* ROS generation in bacterial membrane could damage and inhibit the bacteria eventually. As a proof-of-concept, TPA-PyOH was observed to inhibit *S. aureus* biofilm benefited from its efficient adhesion and subsequent PDT treatments. Furthermore, TPA-PyOH exhibited excellent biocompatibility at dark, which also laid a solid foundation for its promising clinical translational applications.

## Data Availability

The original contributions presented in the study are included in the article/Supplementary Material, further inquiries can be directed to the corresponding authors.
