# Supplementary material for: Fast Broad-Spectrum Staining and Photodynamic Inhibition of Pathogenic Microorganisms by a Water-Soluble Aggregation-Induced Emission Photosensitizer
Source: Front Chem. 2021 Nov 2;9:755419. doi: 10.3389/fchem.2021.755419 (PMC8593337; doi:10.3389/fchem.2021.755419)
Supplement: Supplementary file 1 [file DataSheet1.docx]

Supplementary Material

**Fast Broad-Spectrum Staining and Photodynamic Inhibition of Pathogenic Microorganisms by a Water-soluble Aggregation-Induced Emission Photosensitizer**

**Qi Zhou^1,2,†^, Xiaoming Lyu^4,†^, Bing Cao^1,2^, Xueping Liu^1,2^, Jing Liu^1,2^, Jiarui Zhao^1,2^, Siyu Lu^5^, Meixiao Zhan^3*^, Xianglong Hu^1,2*^**

^1^ MOE Key Laboratory of Laser Life Science & Institute of Laser Life Science, College of Biophotonics, South China Normal University, Guangzhou 510631, China

^2^ Guangdong Provincial Key Laboratory of Laser Life Science, & Guangzhou Key Laboratory of Spectral Analysis and Functional Probes, College of Biophotonics, South China Normal University, Guangzhou 510631, China

^3^ Zhuhai Precision Medical Center, Zhuhai People’s Hospital, Zhuhai Hospital Affiliated with Jinan University, Jinan University, Zhuhai, Guangdong 519000, China

^4^ Department of Laboratory Medicine, The Third Affiliated Hospital, Southern Medical University, Guangzhou, China

^5^ Green Catalysis Center, and College of Chemistry, Zhengzhou University, Zhengzhou, 450000, China

*** Correspondence:**Corresponding Author
huxlong@mail.ustc.edu.cn, xlhu@scnu.edu.cn (X. Hu)
zhanmeixiao1987@126.com (M. Zhan)


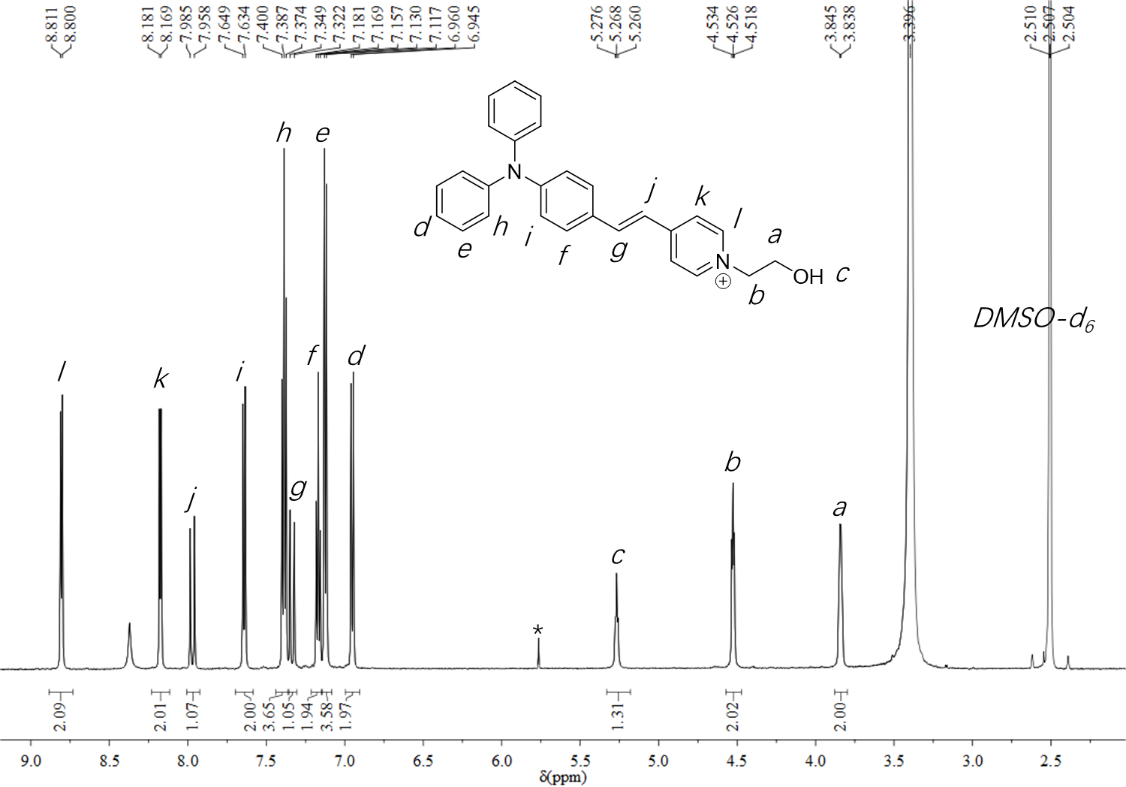


**Supplementary Figure 1.** ^1^H NMR spectrum of TPA-PyOH recorded in DMSO-*d*_6_.

**
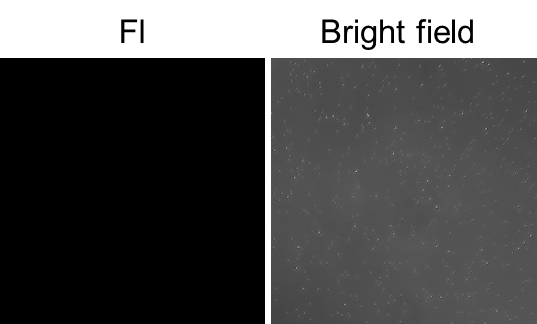
**

**Supplementary Figure 2.** CLSM images (40×) of *S. aureus* in the absence of TPA-PyOH. The image was excited at 488 nm, and the emission was collected at 520-670 nm.


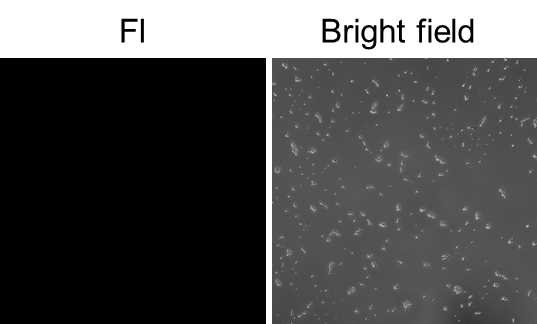


**Supplementary Figure 3.** CLSM images (40×) of *L. monocytogenes* in the absence of TPA-PyOH. The image was excited at 488 nm, and the emission was collected at 520-670 nm.


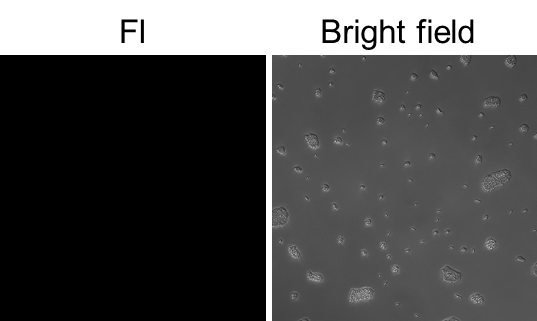


**Supplementary Figure 4.** CLSM images (40×) of *E. coli* in the absence of TPA-PyOH. The image was excited at 488 nm, and the emission was collected at 520-670 nm.


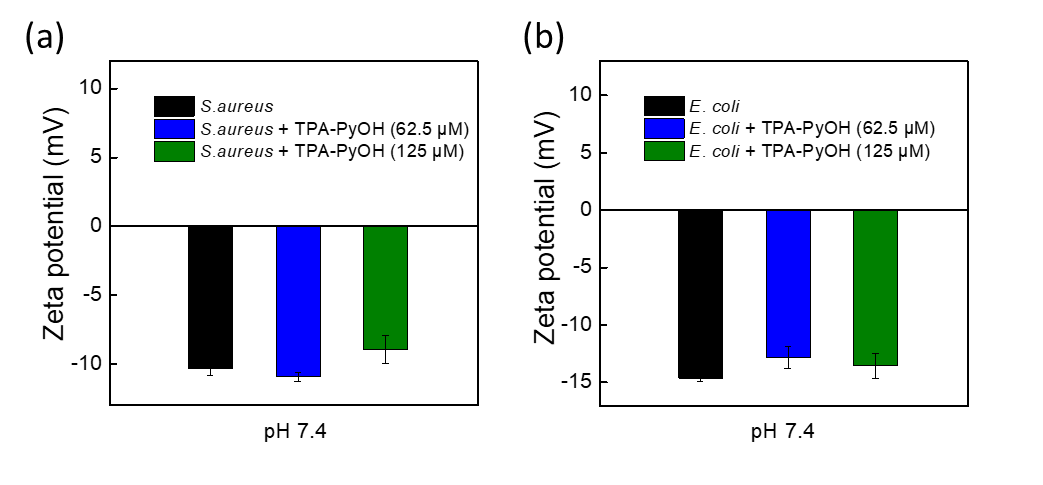


**Supplementary Figure 5.** Zeta potential of *S. aureus* and *E. coli* before and after incubating with TPA-PyOH for 10 min in PBS buffer.


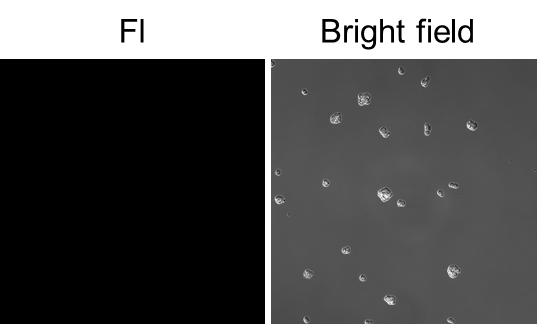


**Supplementary Figure 6.** CLSM images (40×) of *C. albicans* in the absence of TPA-PyOH. The image was excited at 488 nm, and the emission was collected at 520-670 nm.


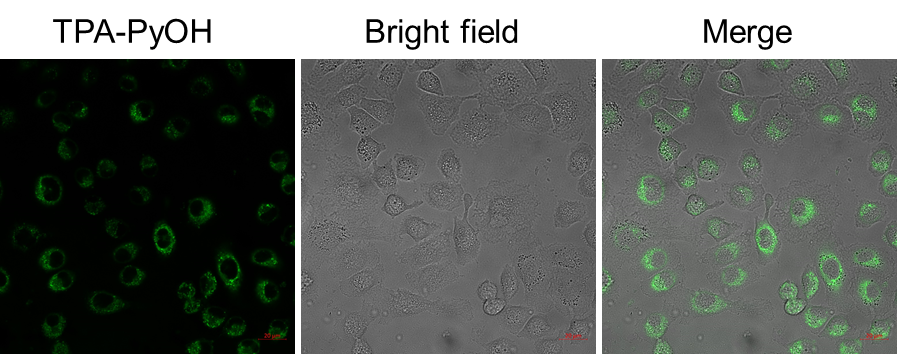


**Supplementary Figure 7.** Wash-free CLSM imaging (40×) of EMT6 cells upon incubation with TPA-PyOH for 4 h without extra washing. The image was excited at 488 nm, and the emission was collected at 520-670 nm. Green pseudo color is used to represent fluorescent signal from TPA-PyOH channel.

**Supplementary Figure 8.** *In vitro* cytotoxicity analysis for TPA-PyOH upon incubation with EMT6 cells for 24 h in dark.
